# Novel approaches to the management of recurrent pregnancy loss: The OPTIMUM (OPtimization of Thyroid function, Thrombophilia, Immunity, and Uterine Milieu) treatment strategy

**DOI:** 10.1002/rmb2.12412

**Published:** 2021-09-14

**Authors:** Keiji Kuroda, Yuko Ikemoto, Takashi Horikawa, Azusa Moriyama, Yuko Ojiro, Satoru Takamizawa, Toyoyoshi Uchida, Shuko Nojiri, Koji Nakagawa, Rikikazu Sugiyama

**Affiliations:** ^1^ Centre for Reproductive Medicine and Implantation Research Sugiyama Clinic Shinjuku Tokyo Japan; ^2^ Department of Obstetrics and Gynaecology Faculty of Medicine Juntendo University Tokyo Japan; ^3^ Departments of Metabolism and Endocrinology Faculty of Medicine Juntendo University Tokyo Japan; ^4^ Medical Technology Innovation Centre Juntendo University Tokyo Japan; ^5^ Clinical Research and Trial Centre Juntendo University Hospital Tokyo Japan

**Keywords:** chronic endometritis, helper‐T cell, hypothyroidism, recurrent pregnancy loss, thrombophilia

## Abstract

**Purpose:**

Does the OPtimization of Thyroid function, Thrombophilia, Immunity, and Uterine Milieu (OPTIMUM) treatment strategy, developed for treating repeated implantation failure (RIF), contribute to improving pregnancy outcomes in patients with a history of recurrent pregnancy loss (RPL)?

**Methods:**

Between 2018 and 2019, women with RPL after two or more clinical pregnancy losses underwent RPL testing. We treated chronic endometritis with antibiotics, high Th1/Th2 cell ratios with vitamin D and/or tacrolimus, overt/subclinical hypothyroidism with levothyroxine, and thrombophilia with low‐dose aspirin. Of 168 consecutive women aged ≤43 years, 115 underwent RPL testing. We compared 100 pregnancies (90 women) and 46 pregnancies (41 women) with and without the OPTIMUM treatment strategy, respectively.

**Results:**

RPL testing identified intrauterine abnormalities in 66 (57.4%), elevated Th1/Th2 cell ratios in 50 (43.5%), thyroid dysfunction in 33 (28.7%), and thrombophilia in 33 (28.7%). The live birth rate in the OPTIMUM group was significantly higher than that in the control group among women aged <40 years (78.1% and 42.3%, respectively; *p* = 0.002), but no significant difference was observed in women aged ≥40 years (55.6% and 30.0%, respectively; *p* = 0.09).

**Conclusions:**

The OPTIMUM treatment strategy improved pregnancy outcomes in patients with not only RIF but also RPL.

## Graphical abstract

The OPtimization of Thyroid function, Thrombophilia, Immunity, and Uterine Milieu (OPTIMUM) treatment strategy was developed for treating repeated implantation failure (RIF). The live birth rate after the OPTIMUM treatment strategy was 78.1% and significantly higher than that in the control group (42.3%) among women aged <40 years (*p* = 0.002).The OPTIMUM treatment strategy contributed to improving pregnancy outcomes in patients with a history of not only RIF but also recurrent pregnancy loss (RPL).

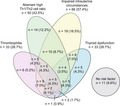

## 1 INTRODUCTION

Pregnancy loss is the most frequent complication during pregnancy.^1^ Yet, ≥50% of women with a history of recurrent pregnancy loss (RPL) have no risk factors.^2^, ^3^, ^4^ Women with unexplained RPL may suffer repeated sporadic miscarriage, but cannot give birth because of risk factors undetectable using common RPL screening.^5^ Although many clinical trials for unexplained RPL have been performed, there remains no established treatment.^6^, ^7^

Pregnancy loss is considered a multifactorial disease, as complex multiple influences including lifestyle habits also affect pregnancy outcomes.^8^, ^9^, ^10^ We previously reported a combination treatment for abnormalities of intrauterine circumstances, immune status, and thyroid function, referred to as the “OPTIMUM” (OPtimization of Thyroid function, Immunity, and Uterine Milieu) treatment strategy, for treatment of repeated implantation failure (RIF) after multiple embryo transfer cycles in assisted reproductive technology (ART) treatment.^11^ In this strategy, we detected and treated the risk factors for reproductive failure including intrauterine disorders, elevated helper‐T (Th)1/Th2 cell ratios, and thyroid dysfunction.

Thyroid dysfunction has been established as a risk factor for RPL.^12^, ^13^ but intrauterine organic abnormalities including endometrial polyps and submucosal myomas remain insufficient evidence.^12^ However, hysteroscopic surgery dramatically improves pregnancy rate, because intrauterine disorders can impair the chance of embryo implantation.^14^, ^15^ Persistent endometrial inflammation (ie, chronic endometritis [CE]) is also not included among the risk factors of RPL because of a lack of reliable evidence. However, CE was recognized in the local endometrium of 24% to 56% of women with a history of RPL.^16^, ^17^, ^18^ Previous research showed that unexplained RPL is involved in an endometrial proinflammatory response with an abnormally increased density of uterine natural killer (uNK) cells and aberrant angiogenesis in endometrial stromal cells^19^, ^20^, ^21^; therefore, CE is a potential risk factor for RPL. The relationship between immunological abnormality and RPL is also unknown. Successful pregnancy requires balancing Th1 and Th2 cells with secretion of pro‐ and anti‐inflammatory cytokines, respectively; thus, perturbations in the Th1/Th2 cell ratio with Th1 bias can cause not only implantation failure but also pregnancy loss.^22^, ^23^ Local and systemic abnormal inflammatory reactions are linked to unexplained RPL. Furthermore, in our previous study of the OPTIMUM treatment strategy, we examined and treated thrombophilia with low‐dose aspirin, because the prevalence of thrombophilia is relatively high in women with an RIF history.^24^, ^25^ Therefore, the OPTIMUM treatment strategy can cover various risk factors for pregnancy loss.

In this study, we analyzed the prevalence of impaired intrauterine circumstances, elevated Th1/Th2 cell ratios, thyroid dysfunction, and thrombophilia, and the prognosis of clinical pregnancy in women with a history of RPL after the OPTIMUM treatment strategy.

## 2 MATERIALS AND METHODS

### 2.1 Patient selection

**FIGURE 1:**
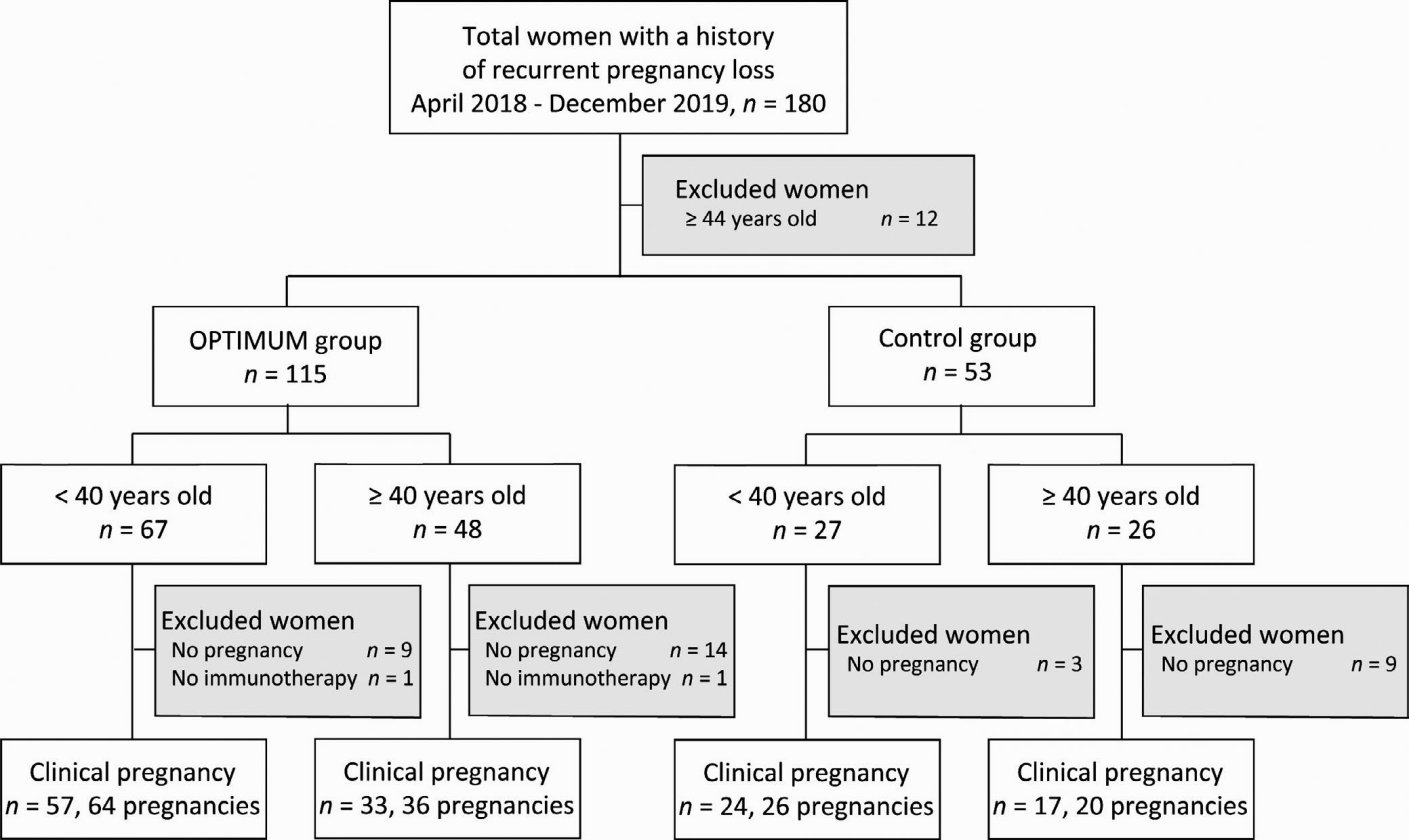
Flowchart of patient selection. Of 180 women with recurrent pregnancy loss (RPL), we recruited 168 women, including 115 women who underwent our RPL testing (OPTIMUM group) and 53 women without RPL testing (control group); 12 women aged ≥44 years were excluded. To analyze the efficacy of the OPTIMUM treatment strategy, 100 pregnancies in 90 women in the OPTIMUM group and 46 pregnancies in 41 women in the control group were recruited

This study is a retrospective cross‐sectional study. A total of 180 consecutive women with a history of RPL after two or more clinical pregnancy losses visited the Sugiyama Clinic Shinjuku from April 2018 to December 2019. After excluding 12 women aged ≥44 years, 168 women were recruited (Figure 1). To detect the prevalence of risk factors for RPL, 115 women including 67 aged <40 years and 48 aged ≥40 years underwent our RPL testing for the OPTIMUM treatment strategy in our clinic. The remaining 53 women without RPL testing including examinations for thyroid function and thrombophilia were also recruited as the control group. In the control group, some of the patients did not desire RPL testing or some doctors did not suggest RPL testing.

In the OPTIMUM group, 102 clinical pregnancies were confirmed in 92 women. We excluded two women who did not want to undergo our immunotherapy, tacrolimus; therefore, 100 pregnancies in 90 women were recruited for analysis of the therapeutic efficacy of the OPTIMUM treatment strategy. In the control group, 46 clinical pregnancies were confirmed in 41 women. Therefore, we compared the pregnancy prognosis in the OPTIMUM group with that of the control group. An intrauterine gestational sac by transvaginal ultrasound was used to diagnose clinical pregnancy. Pregnancy loss was defined as a loss of clinical pregnancy, not a biochemical pregnancy. Infertility was defined as the failure to achieve clinical pregnancy after unprotected intercourse for 12 months or longer.

We compared women with a successful live birth (live birth group) and those with a pregnancy loss (miscarriage group) in their first pregnancies among 90 women who conceived after the OPTIMUM treatment strategy. To analyze the predictive factors in 113 women, including those unable to conceive for 6 to 14 months after the OPTIMUM treatment strategy, we also compared women who had a successful live birth within two pregnancies (success group) and those with no pregnancy or pregnancy loss (failure group). This study was approved by the local ethics committee of Juntendo University, Faculty of Medicine (No. 14–103) and Sugiyama Clinic (No. 18–002).

### 2.2 OPTIMUM treatment strategy protocol

**FIGURE 2:**
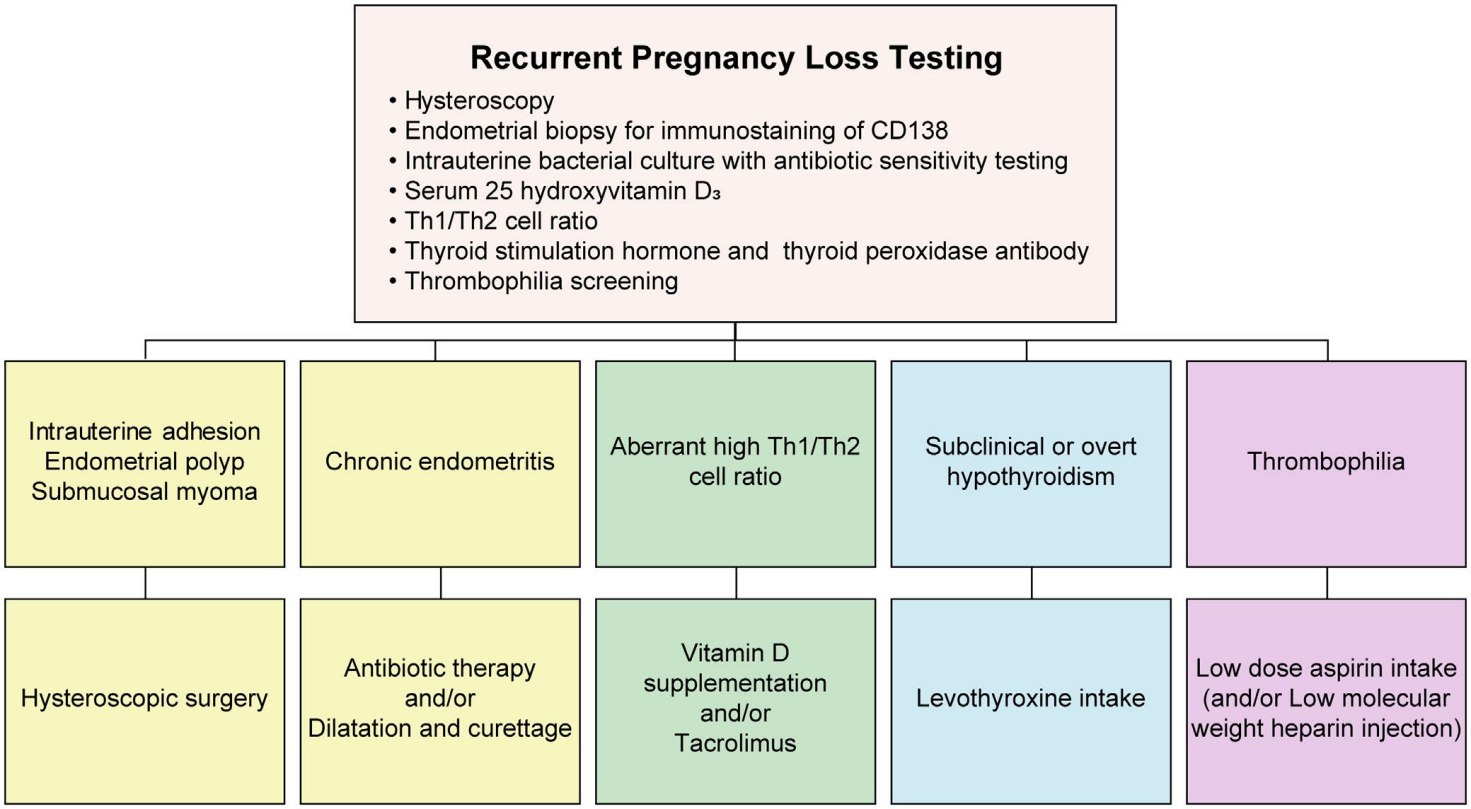
Recurrent pregnancy loss testing and treatment for risk factors of pregnancy loss. Recurrent pregnancy loss (RPL) testing included a hysteroscopy, endometrial biopsy for CD138 immunostaining and bacterial culture, and blood testing for 25‐hydroxyvitamin D_3_, interferon‐γ–producing helper‐T (Th1) cell, interleuking‐4‐producing helper‐T (Th2) cell, thyroid function, and thrombophilia. We treated intrauterine organic disorders with hysteroscopic surgery, chronic endometritis with antibiotics and/or dilatation and curettage, high Th1/Th2 cell ratios with vitamin D supplementation and/or tacrolimus, overt or subclinical hypothyroidism with levothyroxine and thrombophilia with low‐dose aspirin and/or low‐molecular‐weight heparin [Colour figure can be viewed at wileyonlinelibrary.com]

The design of OPTIMUM treatment strategy was described previously^11^ (Figure 2). Our RPL testing consisted of the following: hysteroscopy; endometrial biopsy for CD138 immunostaining and intrauterine bacterial culture; measurement of serum levels of 25‐hydroxyvitamin D_3_ (25OHVD), interferon (IFN)‐γ–producing Th cell (Th1 cell) and interleukin (IL)‐4‐producing Th cell (Th2 cell); testing for thyroid function including thyroid‐stimulating hormone (TSH) levels and thyroid peroxidase antibody (TPOAb); and thrombophilia screening for antiphospholipid syndrome (APS), protein C and S deficiency and factor XII deficiency. We did not perform preimplantation genetic testing for aneuploidy (PGT‐A) and structural rearrangements (PGT‐SR) or endometrial microbiome analysis.

### 2.3 Medical interview for lifestyle habits

Pregnancy loss is caused by multiple environmental, genetic, and lifestyle factors.^8^, ^9^ Risk factors that increase the rate of pregnancy loss 1.5‐ to 2‐fold include smoking 10 to 20 or more cigarettes daily,^26^, ^27^ daily caffeine intake of two to three cups of coffee,^28^ drinking alcohol twice a week or more,^29^ and obesity with a body mass index >30 kg/m^2^.^30^ Thus, we confirmed the women's lifestyle habits and recommended adjustments such as cessation of smoking, caffeine intake, and drinking as well as diet and moderate exercise for obesity. The experience of pregnancy loss is often physical burden, leading to depression, anxiety disorders, and insomnia.^31^, ^32^, ^33^ Maternal stress is involved in an increased risk of pregnancy loss.^34^, ^35^ Therefore, counseling was provided as necessary to relieve their stress and anxiety.

### 2.4 Tests and treatment for intrauterine circumstances

To confirm intrauterine milieu, we performed a hysteroscopy and endometrial biopsy for CE. When intrauterine diseases including submucosal myomas, endometrial polyps, and intrauterine adhesion were detected by hysteroscopy, we performed hysteroscopic surgery first. On the day of surgery, all women underwent endometrial sampling for intrauterine bacterial culture with antibiotic sensitivity testing using endometrial suction curette (Pipet Curet; Fuji Medical Corporation, Tokyo, Japan) after the vagina was washed sufficiently with physiological saline to prevent sample contamination. All intrauterine disorders and typical CE findings such as an erythrogenic surface, stroma, and micropolyps^36^ were removed using a monopolar resecting loop (Olympus, Tokyo, Japan) without applying electrodes, as described previously.^37^ The specimens were fixed in 10% formaldehyde for histological examination with CD138 immunohistochemistry staining. We sent the samples to BML, Inc. (Tokyo, Japan) for both CD138 immunostaining and bacterial cultures. Pathologists stained the specimens using anti‐CD138 antibodies (M7228; Dako, Agilent Technologies Japan, Ltd., Tokyo, Japan) and counted the CD138‐positive plasmacytes in 10 nonoverlapping random stromal areas visualized at 400‐fold magnification (BML, Inc.); CE was diagnosed with the presence of five or more CD138‐positive cells. In our previous study, most CE in women with endometrial polyps were cured by polypectomy without antibiotic therapy.^37^ Unnecessary antibiotic treatment decreased the CE recovery rates by polypectomy and pregnancy outcomes after surgery.^37^ Therefore, CD138 immunostaining and bacterial cultures were reexamined without antibiotics during the luteal phase in the next menstruation cycle after surgery.

Our treatment protocol for CE without intrauterine disorders was described previously.^38^ Women underwent endometrial sampling during the luteal phase. When CE was diagnosed, patients received oral doxycycline (Vibramycin^®^ tablets; Pfizer Japan Inc., Tokyo, Japan), 100 mg twice a day for 2 weeks as the first choice. If CE was not cured and specific bacteria were detected (except for *Lactobacillus* spp. or *Bifidobacterium* spp.), second‐line therapy consisted of bacterium‐sensitive antibiotics for 2 weeks, based on the results of the antibiotic sensitivity testing. If CE was not cured without specific bacteria, we administered a combination of amoxicillin (Sawacillin^®^ tablets, 250 mg; LTL Pharma Co., Ltd.), azithromycin (Azithromycin tablets, 250 mg; Fuji Pharma Co., Ltd.), metronidazole (Flagyl^®^, 250 mg; Shionogi & Co., Ltd.), and antibiotic‐resistant lactic acid bacteria (Biofermin‐R^®^ tablets, 6.0 mg; Biofermin pharmaceutical Co., Ltd.) twice daily for 2 weeks. When CE was not cured by antibiotic treatment, dilatation and curettage (D&C) was performed to remove the endometrium with plasmacytes.

### 2.5 Tests and treatment for Th1/Th2 cell balance

Immune testing consisted of measuring serum 25OHVD, IFN‐γ, and IL‐4 using SRL Inc. (Tokyo, Japan) as described previously.^39^, ^40^ Vitamin D deficiency, insufficiency, and sufficiency were diagnosed as 25OHVD levels of <12, between ≥12 and <30, and ≥30 ng/ml, respectively.^41^ Th1 and Th2 cell levels were measured as CD4^+^ T lymphocytes with IFN‐γ without IL‐4 and CD4^+^ T lymphocytes with IL‐4 without IFN‐γ, respectively.

Vitamin D insufficiency and deficiency were treated with vitamin D supplementation (Sugiyama Clinic Original Vitamin D Supplement, Calinesse, Tokyo, Japan) containing vitamin D_3_ (cholecalciferol). Vitamin D was supplemented at 1000 or 2000 IU daily for 25OHVD levels of ≥20 and <30 ng/ml or <20 ng/ml, respectively, based on our previous report.^39^ Based on the previous study, the definition of high Th1/Th2 cell ratios was determined to be >10.3.^40^ Elevated Th1/Th2 cell ratios can be attenuated by vitamin D supplementation^39^; therefore, when the women had high Th1/Th2 cell ratios and low 25OHVD levels, the serum levels of 25OHVD, Th1, and Th2 cells were retested after 3 months or more of vitamin D intake. When their 25OHVD levels were <30 ng/ml, the daily dose of vitamin D supplementation was increased by a further 1000 IU. When high Th1/Th2 cell ratios could not be controlled, we treated them using an immunosuppressive drug, tacrolimus (Prograf^®^ capsules, 1 mg; Astellas Pharma), as described previously.^40^, ^42^ Women with RPL who had Th1/Th2 cell ratios of 10.3–12.9, 13.0–15.7, and ≥15.8 were treated with 1, 2, and 3 mg of tacrolimus daily, respectively, from the day of positive pregnancy test (4–5 weeks of gestation). In some women with a history of RIF, the intake of tacrolimus was initiated from 1 day before the day of embryo transfer, as in our previous trial.^11^ In women with aberrant elevated Th1 cell levels (≥28.8), the dosage of tacrolimus was increased by a further 1 mg.^42^

### 2.6 Tests and treatment for thyroid function

To examine thyroid function, we measured serum TSH and TPOAb levels using a commercial electrochemiluminescence immunoassay (normal range: 0.56–4.30 μIU/ml, Roche Diagnostics) and an enzyme‐linked immunosorbent assay (normal range: <16.0 IU/ml; Roche Diagnostics), respectively. The treatment threshold for thyroid abnormalities using levothyroxine in the patients with RPL was TSH ≥2.5 μIU/ml. All women with levothyroxine treatment maintained TSH levels <2.5 μIU/ml and continued until live birth.

### 2.7 Tests and treatment for thrombophilia

Thrombophilia tests consisted of measuring serum levels of protein C and S activities, factor XII, antiphospholipid antibodies including lupus anticoagulant, anticardiolipin antibodies including IgG and IgM and anti‐β2‐GP1 antibodies including IgG and IgM. When positive antiphospholipid antibody was confirmed, the detected antibody was remeasured 3 months later and the decision to administer low‐dose aspirin (Bafferin Combination Tablet A81, 81 mg; Eisai Co., Ltd.) was made. Treatment with low‐dose aspirin was started from the day of the positive pregnancy test. In some women with a history of RIF, daily aspirin intake was initiated from 10 days after ovulation or the start of progesterone administration in embryo transfer cycles, as in our previous trial.^11^ We administered low‐molecular‐weight heparin to only one woman with APS who had a clinical pregnancy loss with normal karyotype even with treatment with low‐dose aspirin.

### 2.8 Treatment for women without risk factors

When any risk factors, except for vitamin D insufficiency, were not detected after RPL testing, we recommended supplementation of 30 mg dydrogesterone tablets (Duphastone 5 mg, Abbott Japan LLC, Tokyo, Japan) three times daily until 12 weeks of gestation. Deficient or inadequate progesterone secretion during the luteal phase is associated with pregnancy loss,^43^ and unexplained RPL is involved in perturbed decidual change of the endometrium.^44^, ^45^ Delayed implantation timing of an embryo is also linked to an increased risk of subsequent pregnancy loss rates.^46^ Therefore, luteal support with progesterone treatment has a potential therapeutic effect on inducing decidualization of the endometrium and optimizing the timing of implantation, leading to pregnancy loss prevention.

### 2.9 Statistical analysis

All statistical analyses were performed using Statistical Analysis System version 9.4 (SAS Institute, Cary, NC, USA). Statistical significance was calculated using the *t* test for differences in continuous variables, and chi‐square and Fisher's exact tests were used as appropriate to test the statistical significance of the categorical variables. To identify predictive factors that affect pregnancy outcomes after the OPTIMUM treatment strategy in women with RPL, we constructed a multivariable logistic regression model to determine the independent factors while controlling for confounders. Odds ratios (ORs) and their 95% confidence intervals (CIs) were computed. The level of significance was defined as *p* < 0.05.

## 3 RESULTS

### 3.1 Prevalence of risk factors for pregnancy loss

**FIGURE 3:**
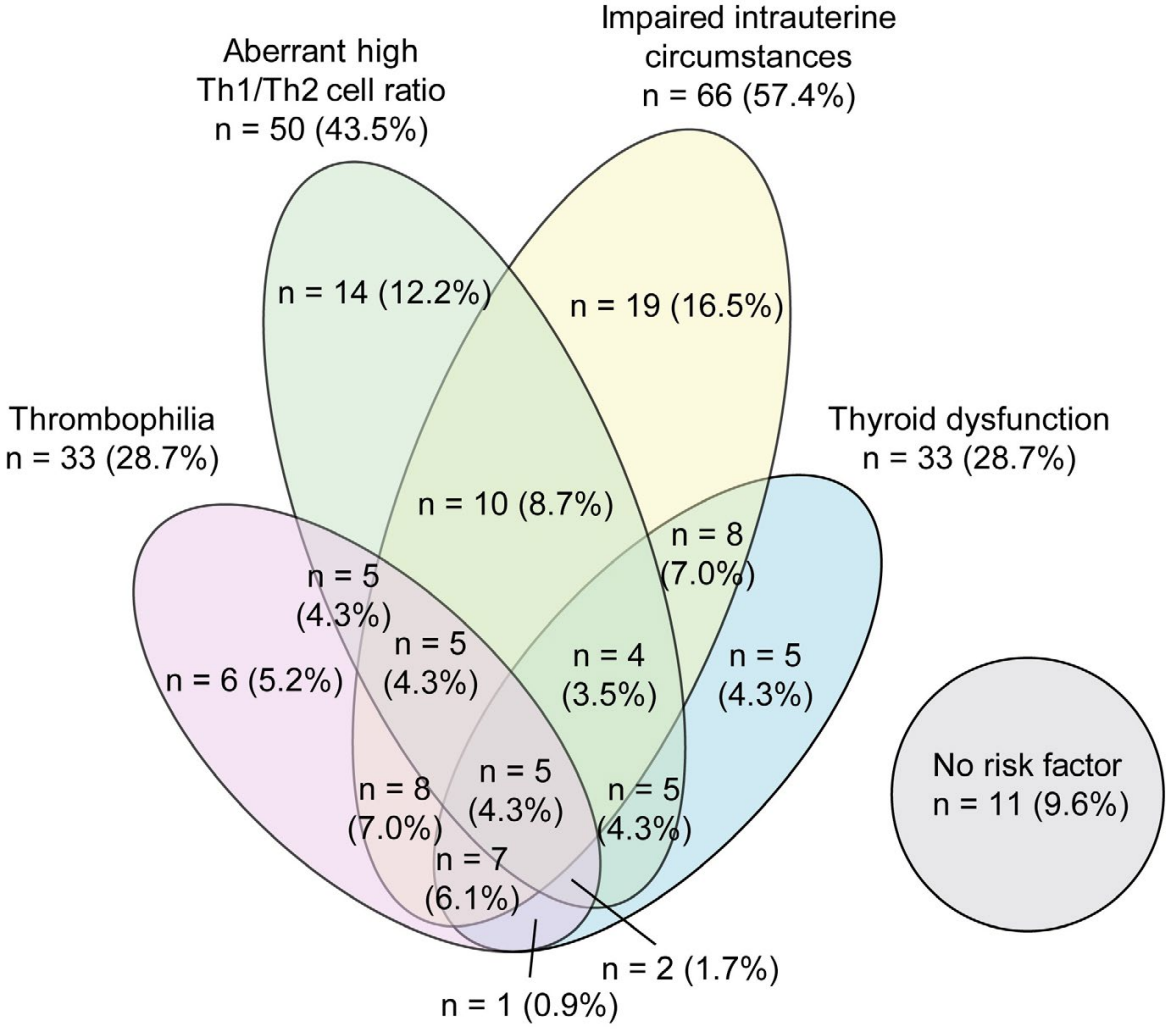
Prevalence of risk factors for pregnancy loss. Venn diagram showing the number of women with thyroid dysfunction, impaired intrauterine circumstances, high Th1/Th2 cell ratios, thyroid, and thrombophilia [Colour figure can be viewed at wileyonlinelibrary.com]

Table S1 shows the characteristics of the women with a history of RPL who underwent the OPTIMUM treatment strategy. The prevalence of risk factors for pregnancy loss is shown in Table 1. The prevalence of intrauterine abnormalities was 61.2% (41 women) and 52.1% (25 women) among women aged <40 and ≥40 years, respectively. Of the 66 women with impaired intrauterine circumstances, 90.9% (60 women) had CE. With regard to immunological status, ≥90% of women with RPL had vitamin D insufficiency or deficiency. The prevalence of an aberrant high Th1/Th2 cell ratio was 43.3% (29 women) and 43.8% (21 women) among women aged <40 and ≥40 years, respectively. Thyroid disorders including subclinical or overt hypothyroidism were recognized in 34.3% (23 women) and 29.2% (14 women) of women aged <40 and ≥40 years, respectively. Furthermore, thrombophilia was found in 37.3% (25 women) and 29.2% (14 women) of the women aged <40 and ≥40 years, respectively, including 11 women diagnosed with APS. Relationships among impaired intrauterine circumstances, elevated Th1/Th2 cell ratios, thyroid disorders, and thrombophilia are shown in Figure 3. The RPL testing showed that 60 (52.2%) of the RPL women had two or more risk factors, including 5 (4.3%) who had all four risk factors. The relationships among four risk factors for pregnancy loss were irregular and complicated. However, there were no risk factors in 11 women (9.6%).

**TABLE 1:** Prevalence of risk factors for pregnancy loss

	< 40 years, *n* = 67	≥ 40 years, *n* = 48
Intrauterine circumstance		
Normal, *n* (%)	26 (38.8)	23 (47.9)
Chronic endometritis, *n* (%)	38 (57.0)	22 (46.0)
Endometrial polyp, *n* (%)	3 (4.5)	4 (8.3)
Intrauterine adhesion, *n* (%)	3 (4.5)	1 (2.1)
Submucosal myoma, *n* (%)	1 (1.5)	0 (0)
Total women with impaired intrauterine circumstance, *n* (%)^a^	41 (61.2)	25 (52.1)
Immunological tolerance		
Vitamin D		
25‐hydroxyvitamin D3, ng/ml, mean ±SD	20.2 ± 7.1	18.6 ± 6.2
Sufficiency, *n* (%)	5 (7.5)	4 (8.3)
Insufficiency, *n* (%)	56 (83.6)	39 (81.3)
Deficiency, *n* (%)	6 (9.0)	5 (10.4)
Total women with lack of vitamin D, *n* (%)	62 (92.5)	44 (91.7)
Helper T cells		
Th1 cell, %, mean ± SD	24.9 ± 9.2	21.7 ± 7.1
Th2 cell, %, mean ± SD	2.6 ± 0.9	2.4 ± 1.0
Th1/Th2 cell ratio, %, mean ± SD	10.9 ± 6.5	10.6 ± 5.4
Total women with aberrant high Th1/Th2 cell ratio, *n* (%)	29 (43.3)	21 (43.8)
Thyroid function		
Subclinical hypothyroidism, *n* (%)	20 (29.9)	12 (25.0)
Overt hypothyroidism, *n* (%)	2 (3.0)	2 (4.2)
Hyperthyroidism, *n* (%)	1 (1.5)	0 (0)
Thyroid peroxidase antibody‐positive, *n* (%)^b^	11 (16.4)	7 (14.6)
Total women with thyroid dysfunction, *n* (%)	23 (34.3)	14 (29.2)
Thrombophilia		
Lupus anticoagulant positive, *n* (%)	2 (3.0)	0 (0)
Anticardiolipin antibody (IgG, IgM) positive, *n* (%)	4 (6.0)	4 (8.3)
Anti‐β2‐GP1 antibody (IgG, IgM) positive, *n* (%)	1 (1.5)	0 (0)
Total women with antiphospholipid syndrome, *n* (%)	7 (10.4)	4 (8.3)
Protein C deficiency, *n* (%)	1 (1.5)	1 (2.1)
Protein S deficiency, *n* (%)	2 (3.0)	3 (6.3)
Factor XII deficiency, *n* (%)	18 (26.9)	7 (14.6)
Total women with thrombophilia, *n* (%)^c^	25 (37.3)	14 (29.2)

^a^ Four women aged <40 years and two aged ≥40 years had two intrauterine disorders.

^b^ Eighteen women with thyroid peroxidase antibody‐positive included 4 and 14 women with overt and subclinical hypothyroidism, respectively.

^c^ Three women aged <40 years and one aged ≥40 years had two thrombophilia.

### 3.2 Treatment for CE and aberrant high Th1/Th2 ratios

Of the 60 women with CE, CE was detected in five and two women after hysteroscopic polypectomy and adhesiolysis, respectively. After surgery, recovery from CE was confirmed with reexamination of endometrial biopsy without antibiotics in five women. In two women, CE was cured by treatment using bacterium‐sensitive antibiotics, as in our previous report.^37^ Of the remaining 53 women without intrauterine disorders, 41 (68.3%) and 11 (18.3%) recovered from CE in the first and second cycles of antibiotic therapy, respectively. One woman (1.7%) without recovery from CE after two cycles of antibiotic therapy underwent D&C and was cured of CE. Finally, all women in our study recovered from CE.

Of the 50 women with high Th1/Th2 cell ratios, 46 (92.0%) had a lack of vitamin D. In 28 women, 3 to 6 months after starting vitamin D intake at 1000–2000 IU daily, a retest of the Th1/Th2 cell ratio and 25OHVD level showed an increase in 25OHVD from 18.1 ± 5.0 ng/ml to 31.2 ± 7.3 ng/ml (*p* < 0.001) and a decrease in the Th1/Th2 cell ratio from 16.9 ± 5.9 to 13.1 ± 4.8 (*p* = 0.007). The Th1/Th2 cell ratio was reduced in 25 women (89.3%) including 10 women (35.7%) with normalized the ratio (<10.3). For the 30 women with clinical pregnancy with a high Th1/Th2 cell ratio even after vitamin D supplementation, we recommended daily administration of tacrolimus beginning after a positive pregnancy test. No patients had any side effects from vitamin D and tacrolimus treatment.

### 3.3 Pregnancy outcomes after OPTIMUM treatment strategy

To investigate the efficacy of the OPTIMUM treatment strategy for preventing pregnancy loss in women with RPL, we compared pregnancy outcomes between those who received the OPTIMUM treatment strategy and those who did not. Table 2 summarizes the characteristics of the women with RPL in both the control and OPTIMUM groups. Differences in age, pregnancy history, anti‐Müllerian hormone (AMH) levels, prevalence of infertility, and pregnancy procedures were not significant in both groups. With regard to pregnancy outcomes, the live birth rate in the OPTIMUM group was significantly higher than that in the control group among women aged <40 years (78.1% and 42.3% per pregnancy, respectively; *p* = 0.002). In women aged ≥40 years, the live birth rate was higher in the OPTIMUM group than that in the control group; however, there was no significant difference (55.6% and 30.0% per pregnancy, respectively; *p* = 0.09). Of 59 women with miscarriage, 33 (55.9%) underwent chromosome analysis of products of conception. The rate of normal karyotype in women aged <40 years in the control group was relatively higher (40.0%), yet there were no significant differences between the control and OPTIMUM groups in both patients aged <40 and ≥40 years (*p* = 0.34 and 1.00, respectively). Finally, 87.7% (50/57 women) and 60.6% (20/33 women) of the patients aged <40 and ≥40 years, respectively, had a child birth in one or two clinical pregnancies after the OPTIMUM treatment strategy.

**TABLE 2:** Clinical characteristics and pregnancy outcomes in control and OPTIMUM groups

	< 40 years			≥ 40 years		
	Control *n* = 24	OPTIMUM *n* = 57	*p*‐value	Control *n* = 17	OPTIMUM *n* = 33	*p*‐value
Age, years, mean ±SD (range)	35.8 ± 2.8 (30−39)	35.1 ± 3.4 (26−39)	0.40	41.5 ± 1.1 (40−43)	41.2 ± 1.2 (40−43)	0.36
Pregnancy history, median (range)						
Gravida	3 (2−6)	2 (2−5)	0.22	3 (2−6)	3 (2−9)	0.06
Parity	0 (0−2)	0 (0−1)	0.47	0.5 (0−1)	0 (0−1)	0.08
No. of clinical pregnancy losses	2 (2–5)	2 (2−5)	0.63	3 (2–5)	2 (2−9)	0.05
AMH, ng/ml, mean ±SD	3.4 ± 3.0	4.4 ± 3.4	0.55	2.3 ± 2.0	2.0 ± 1.6	0.74
Prevalence of infertility, *n* (%)	19 (79.2)	40 (70.2)	0.59	15 (88.2)	28 (84.8)	1.00
Risk factors for RPL, *n* (%)						
Impaired intrauterine circumstance	−	35 (61.4)	−	−	16 (48.5)	−
Aberrant high Th1/Th2 cell ratio	−	24 (42.1)	−	−	13 (39.4)	−
Thyroid disorder	−	21 (36.8)	−	−	11 (33.3)	−
Thrombophilia	−	23 (40.4)	−	−	11 (33.3)	−
Procedures of pregnancy, *n* (%)^a^	*n* = 26	*n* = 64		*n* = 20	*n* = 36	
Intercourse	5 (19.2)	17 (29.8)	0.53	2 (10.0)	0 (0)	0.12
Intrauterine insemination	0 (0)	3 (5.3)		0 (0)	0 (0)	
ART treatment	21 (80.8)	44 (64.9)		18 (90.0)	36 (100)	
Pregnancy outcome, *n* (%)						
Live birth rate (/pregnancy)	11 (42.3)	50 (78.1)	0.002*	6 (30.0)	20 (55.6)	0.09
Miscarriage rate (/pregnancy)	15 (57.7)	14 (21.9)		14 (70.0)	16 (44.4)	
Cumulative live birth rate (/patient)	11 (45.8)	50 (87.7)	<0.001*	6 (35.3)	20 (60.0)	0.14
Chromosome analysis of POC, *n* (%)	*n* = 10	*n* = 7		*n* = 8	*n* = 8	
Normal karyotype	4 (40.0)	1 (14.3)	0.34	2 (25.0)	1 (12.5)	1.00
Aneuploid	6 (60.0)	6 (85.7)		6 (75.0)	7 (87.5)	

Abbreviations: AMH, anti‐Müllerian hormone; ART, assisted reproductive technology; POC, products of conception; RPL, recurrent pregnancy loss; SD, standard deviation.

^a^ In the pregnancy prognosis, we compared between 46 clinical pregnancies of 41 women in the control group and 100 pregnancies of 90 women in the OPTIMUM group.

* *p* < 0.05.

### 3.4 Predictive factors for pregnancy outcomes after the OPTIMUM treatment strategy

After the OPTIMUM treatment strategy, 87.7% of the women aged <40 years who suffered from multiple pregnancy losses had live birth; however, the remaining 12.3% of them ended in pregnancy loss. To identify the predictive factors for the therapeutic efficacy of the OPTIMUM treatment strategy, we compared the 62 women who had successfully childbirth (live birth group) and the 28 women who unfortunately ended in pregnancy loss (miscarriage group) in the first clinical pregnancy (Table 3). Women in the live birth group were significantly younger than those in the miscarriage group (36.5 ± 4.0 and 39.1 ± 3.5 years, respectively, *p* = 0.002). Intriguingly, the prevalence of infertility in the miscarriage group was strikingly high at 96.4% compared with 66.1% in the live birth group (*p* = 0.001). There were no significant differences in AMH levels, number of past pregnancy losses, history of live birth, or risk factors of pregnancy loss, including abnormalities of intrauterine circumstances, Th cell balance, thyroid function, and thrombophilia. Univariate analysis also demonstrated that the predictive factors for successful childbirth after the OPTIMUM treatment strategy were younger age (OR = 1.22, 95% CI = 1.06–1.40) and no infertility (OR = 13.83, 95% CI = 1.76–108.95).

**TABLE 3:** Predictive factors for therapeutic effects of OPTIMUM treatment strategy in the women with clinical pregnancy

	Live birth group^a^ *n* = 62	Miscarriage group^a^ *n* = 28	*p*‐value	Univariate analysis OR (95%CI)	Multivariate analysis OR (95%CI)
Age, years, mean ±SD (range)	36.5 ± 4.0 (26−43)	39.1 ± 3.5 (29−43)	0.002*	1.22 (1.06−1.40)	1.23 (0.98−1.53)
AMH, ng/ml, mean ±SD	3.8 ± 3.2	2.5 ± 2.5	0.10	0.68 (0.43−1.08)	1.13 (0.61−2.12)
Prevalence of infertility, *n* (%)	41 (66.1)	27 (96.4)	0.001*	13.83 (1.76−108.95)	8.77 (0.96−80.45)
No of past pregnancy losses, median (range)	2 (2−5)	2 (2−9)	0.30	0.57 (0.20−1.63)	0.44 (0.11−1.86)
Past history of live birth, *n* (%)	14 (22.6)	3 (10.7)	0.25	0.41 (0.11−1.57)	0.25 (0.03−1.88)
Risk factors for RPL					
Impaired intrauterine circumstance, *n* (%)	35 (56.5)	16 (57.1)	1.00	1.03 (0.42−2.53)	1.21 (0.33−4.36)
Aberrant high Th1/Th2 cell ratio, *n* (%)	26 (41.9)	11 (39.3)	1.00	0.90 (0.36−2.23)	0.66 (0.18−2.45)
Thyroid disorder, *n* (%)	19 (30.7)	13 (46.4)	0.16	1.96 (0.78−4.91)	1.99 (0.62−6.41)
Thrombophilia, *n* (%)	21 (33.9)	13 (46.4)	0.35	1.69 (0.68−4.20)	2.27 (0.68−7.57)

Abbreviations: AMH, anti‐Müllerian hormone; CI, confidence interval; OR, odds ratio; RPL, recurrent pregnancy loss; SD, standard deviation.

^a^ To identify the predictive factors for the therapeutic efficacy of the OPTIMUM treatment strategy, we compared the 62 women who had successfully childbirth (live birth group) and the 28 women who ended in pregnancy loss (miscarriage group) in the first clinical pregnancy after the OPTIMUM treatment strategy.

* *p* < 0.05.

The OPTIMUM treatment strategy is the treatment procedure not only for RPL but also for infertility.^11^ However, unfortunately, 23 women could not reach clinical pregnancy for 6–14 months after the OPTIMUM treatment strategy. Therefore, we also analyzed the predictive factors in 113 women including 70 women who had a successful live birth within two pregnancies (success group) and 43 women with no pregnancy or with pregnancy loss (failure group; Table 4). The women in the success group were significantly younger and had higher AMH levels as compared with those in the failure group. Univariate analysis showed that the predictive factors for the success of the OPTIMUM treatment strategy were younger age (OR = 1.25, 95% CI = 1.11–1.41), high AMH level (OR = 0.65, 95% CI = 0.44–0.95), and no infertility (OR = 3.84, 95% CI = 1.41–10.45). In the multivariate analysis, the predictive factors were younger age (OR = 1.22, 95% CI = 1.04–1.43) and ART treatment (OR = 0.19, 95% CI = 0.05–0.73).

**TABLE 4:** Predictive factors for therapeutic effects of OPTIMUM treatment strategy in the women with and without clinical pregnancy

	Success group^a^ *n* = 70	Failure group^a^ *n* = 43	*p*‐value	Univariate analysis OR (95%CI)	Multivariate analysis OR (95%CI)
Age, years, mean ± SD (range)	36.7 ± 4.0 (26−43)	39.6 ± 3.1 (29−43)	<0.001*	1.25 (1.11−1.41)	1.22 (1.04−1.43)
AMH, ng/ml, mean ± SD	3.6 ± 3.0	2.4 ± 2.9	0.03*	0.65 (0.44−0.95)	0.85 (0.53−1.35)
Prevalence of infertility, *n* (%)	49 (70.0)	37 (86.0)	0.07	3.84 (1.41−10.45)	3.85 (0.96−15.45)
No of past pregnancy losses, median (range)	2 (2−5)	2 (2−9)	0.58	0.80 (0.35−1.79)	0.79 (0.27−2.33)
Past history of live birth, *n* (%)	15 (21.4)	7 (16.3)	0.63	0.64 (0.24−1.67)	0.54 (0.15−1.97)
Risk factors for RPL					
Impaired intrauterine circumstance, *n* (%)	39 (55.7)	27 (62.8)	0.56	1.20 (0.56−2.54)	1.21 (0.46−3.22)
Aberrant high Th1/Th2 cell ratio, *n* (%)	29 (41.4)	19 (44.2)	0.85	1.05 (0.50−2.22)	1.06 (0.40−2.82)
Thyroid disorder, *n* (%)	21 (30.0)	16 (37.2)	0.54	1.23 (0.56−2.72)	1.16 (0.43−3.10)
Thrombophilia, *n* (%)	25 (35.7)	14 (32.6)	0.84	1.07 (0.49−2.32)	1.30 (0.47−3.56)
Fertility treatment					
Non‐ART treatment, *n* (%)	19 (27.1)	16 (37.2)	0.30	Reference	Reference
ART treatment, *n* (%)	51 (72.9)	27 (62.8)		0.97 (0.43−2.15)	0.19 (0.05−0.73)

Abbreviations: AMH, anti‐Müllerian hormone; ART, assisted reproductive technology; CI, confidence interval; OR, odds ratio; RPL, recurrent pregnancy loss; SD, standard deviation.

^a^ Success group includes women who succeeded childbirth in the first pregnancy after OPTIMUM treatment strategy and failure group involves women who ended in no pregnancy or pregnancy loss at the first pregnancy.

* *p* < 0.05.

## 4 DISCUSSION

Most of the women aged <40 years reached childbirth after the OPTIMUM treatment strategy. Although many risk factors for pregnancy loss, not RPL, have been reported, yet ≥50% of women with RPL have no risk factors in common RPL screening.^3^ In humans, the incidence of pregnancy loss with embryonic chromosomal abnormalities is very high^47^; therefore, various risk factors of pregnancy loss are not statistically included in the factors for RPL.^6^ However, complex multiple influencers of pregnancy loss may trigger RPL as multifactorial disease.^8^, ^9^ In our study, >50% of the women also had two or more risk factors. The only currently established treatments for RPL are levothyroxine supplementation for hypothyroidism and low‐dose aspirin and low‐molecular‐weight heparin for APS.^12^ No efficacious treatment has been found for unexplained RPL.^6^, ^7^ Yet, treatment for risk factors of pregnancy loss, not only RPL, may be necessary while keeping costs low. Our testing in the OPTIMUM treatment strategy costs 50 000–60 000 yen ($US 455–545),^11^ and medical insurance in Japan can cover a part of these examinations including testing for thrombophilia and thyroid function. Therefore, the OPTIMUM treatment strategy does not pose a large financial burden for patients.

Most young women with RPL achieved childbirth under the OPTIMUM treatment strategy. However, in women with advanced aged (≥40 years), this treatment resulted in only 55.6% having a baby in their first pregnancy. Female aging strongly increases the incidence of embryonic chromosomal abnormalities and pregnancy loss.^48^, ^49^ In fact, in our study, the predictive factors of successful live birth in the OPTIMUM treatment strategy included female age. Although we did not perform PGT‐A in this study because it remains in the clinical trial stage in Japan, if PGT‐A can be combined with the OPTIMUM treatment strategy, a further increase in the live birth rate can be expected among women of advanced age.

Infertility is a predictive factor for pregnancy prognosis in the OPTIMUM treatment strategy. In our study, 95.5% (21/22 women) of the women without infertility had their babies in the first pregnancy; therefore, the OPTIMUM treatment strategy has adequate preventive effects on repeated pregnancy losses among women without infertility. However, the miscarriage rate in the women with both RPL and infertility was 39.7% (27/68 women). After women have overcome fertility treatment and successful embryo implantation is confirmed, the experience of fetal loss induces intense psychological stress.^50^ Chronic psychological stress triggers an increased risk of infertility and miscarriage.^34^, ^35^, ^51^, ^52^, ^53^ Stress‐induced anxiety promotes immune responses via a rise in Th1/Th2 cell balance with Th1 bias.^54^ In addition, repeated exposure to semiallogeneic embryos and fetuses may promote systemic immunological rejection. In fact, women with RIF or RPL have a higher Th1/Th2 cell ratio as compared with fertile women.^55^ There is an overlap between immunological factors and other risk factors for RIF and RPL.^56^ Therefore, women with a history of infertility and RPL may be a distinct population who have babies with difficulty. Nevertheless, it is important to assist patients in giving birth before their miscarriage rates are increased with female aging and immunological rejection is induced with repeated reproductive failures.

The univariate analysis showed that advanced age, decreased AMH level, and infertility were associated with no pregnancy or with miscarriage. To shorten the time to live birth, patients need to consider fertility treatment including ART. ART treatment was also detected as the predictive factor in the multivariate analysis but not univariate analysis. In the success group, all women ≥40 years old and/or with AMH <1 ng/mL underwent ART treatment, whereas in the failure group, 27.6% of those ≥40 years old (8/29 women) and 40.0% with an AMH <1 ng/mL (6/15 women) did not undergo ART. In women with advanced age and diminished ovarian reserve, ART treatment is required for successful childbirth. Furthermore, although PGT‐A could not be performed in our study, it should be served as an additional choice in women of advanced age with a history of RPL.

It remains controversial whether RPL is the target of immunotherapy and how many miscarriages immune testing and treatment should be considered.^12^ Our previous study demonstrated that two or more pregnancy losses, but not one, were associated with Th1/Th2 cell imbalance with proinflammatory Th1 bias.^55^ Therefore, women with a history of two or more consecutive miscarriages can undergo testing for Th cell balance. Appropriate vitamin D supplementation for women without a sufficient vitamin D level could improve an aberrantly high Th1 cell level and Th1/Th2 cell ratio.^39^ In our study, vitamin D replacement also decreased the high Th1/Th2 cell ratio in approximately 90% of women, including 35.7% with a normalized Th1/Th2 cell ratio after supplementation. Tacrolimus treatment for women with RPL has been reported,^57^, ^58^ but the evidence is still insufficient. Of the 30 pregnant women with tacrolimus intake for high Th1/Th2 cell ratios even after vitamin D supplementation, 21 (70.0%) reached a live birth in the first pregnancy. Among women aged <40 years, 77.8% (14/18 women) had their babies. Tacrolimus is a safe drug without complications for pregnancy and the fetus when using <3–4 mg per day, which is equivalent to ≤6 ng/ml.^57^ There was also no side effect in the women who supply tacrolimus in our study. Tacrolimus use is one treatment choice for women with a history of RPL and a high Th1/Th2 cell ratio after vitamin D supplementation.

The intrauterine environment is important for the growth of embryos and fetuses. Adverse effects of persistent endometrial inflammatory disease such as CE on female fecundity have been reported.^59^, ^60^, ^61^ Morimune, et al reported a comparison of data from pregnancy outcomes between women with and without CE, finding that the miscarriage rate in women with untreated CE was significantly higher than in women without CE (40.0% and 12.8%, respectively).^62^ Although there have been only few reports on the pregnancy outcomes of women with untreated CE, a relationship between unexplained RPL and local endometrial proinflammatory response with aberrantly increased uNK cells and angiogenesis has been reported.^19^, ^20^, ^21^ Among proinflammatory mediators in women with unexplained RPL, IL33/ST2 activity is disordered in the human endometrium.^63^ Furthermore, nucleotide polymorphisms in NLRP, which is a key gene in the regulation of IL‐1β, are also involved in RPL.^64^ Therefore, local and systemic persistent inflammatory responses with Th1‐related mediators are liked to an increased risk of pregnancy loss.

There is no established treatment for unexplained RPL.^7^ In our study, of the 38 pregnant women without established risk factors for RPL, such as thyroid disorder or thrombophilia, 73.7% (28 women) had their babies in the first pregnancy after the OPTIMUM treatment. Among 24 women aged <40 years, 79.2% (20 women) had a live birth, and the cumulative live birth rate within two pregnancies was 95.8% (23 women). We treated 15 women without any risk factors, including uterine and immune factors, with dydrogesterone during the luteal phase, and vitamin D insufficiency or deficiency was treated with vitamin D supplementation in all 15 women. Progesterone is an essential hormone for decidualization of the human endometrium and pregnancy maintenance. Progesterone can activate the secretion of endometrial cortisone during decidualization of the endometrium, leading to direct and indirect regulation of abnormally increased uNK cells and angiogenesis and optimization of immune tolerance for an embryo.^65^, ^66^, ^67^ In addition, progesterone can reduce contraction of uterine smooth muscle and prostaglandin production.^68^ Therefore, progesterone is a potential inhibitor of pregnancy loss. In a randomized controlled trial of vaginal progesterone suppositories for women with unexplained RPL, no significant difference was found between pregnancy outcomes in women receiving progesterone and those receiving placebo.^69^ However, a systematic review found that synthetic progestogen, dydrogesterone, has a therapeutic effect on unexplained RPL.^70^, ^71^ Systemic treatment with synthetic progestogen may be more effective than vaginal progesterone suppositories. Furthermore, RPL is strongly associated with psychological stress, and treatment with placebo has been shown to relieve stress and decrease the miscarriage rate.^6^ Even if progesterone and vitamin D treatments have no beneficial effects on pregnancy outcomes, mental stress may be reduced by the placebo effects. Treatment using dydrogesterone and vitamin D supplements is inexpensive (300 yen, US dollar 2.7/day for dydrogesterone and 50–100 yen, US dollar 0.5–0.9/d for vitamin D) and has no adverse effects on pregnancy or the fetus. The use of progesterone and vitamin D can be considered as treatment for unexplained RPL.

The OPTIMUM treatment strategy is the first treatment procedure for not only RIF but also RPL. However, the OPTIMUM treatment strategy for RPL is different from that for RIF as follows: the initiation of tacrolimus intake from positive pregnancy test for RPL and 1 day before the day of embryo transfer for RIF; the start of low‐dose aspirin intake from positive pregnancy test for RPL and 10 days after ovulation or the initiation of progesterone administration in embryo transfer cycles; medical interview and recommendation of lifestyle habit improvement for RPL; and progesterone treatment for RPL without any risk factors for pregnancy loss.

This study has some limitations. First, this is a retrospective cohort study. Therefore, the patients in the control group did not desire or were not suggested RPL testing. It might cause a selection bias of this study. Second, there are still no global standard diagnostic criteria for CE. The presence of five or more CD138‐positive plasmacytes in 10 nonoverlapping random stromal areas was defined as CE. The different criteria might result in different outcomes. Third, our recent report showed that, according to data from fertile women, a high Th1/Th2 cell ratio was >11.8.^55^ However, subjects receiving tacrolimus treatment were defined as having a Th1/Th2 cell ratio >10.3 in this study, based on previous data in infertile women with a history of childbirth.^40^ Fourth, data from the chromosomal analysis of the couples were not included in this study.

In conclusion, this is the first report of the OPTIMUM treatment strategy for women with a history of RPL. Combination treatment for abnormalities of intrauterine circumstances, Th1/Th2 cell balance, thyroid function, and thrombophilia can improve pregnancy outcomes in the women with RPL. However, among women with advanced age, diminished ovarian reserve or infertility, fertility treatment including ART should be performed to shorten the time to live birth. In addition, this treatment cannot cover pregnancy loss with embryonic chromosomal abnormalities; therefore, combining the OPTIMUM treatment strategy with PGT‐A may produce further efficacy expected to prevent pregnancy loss in women with advanced age.

## CONFLICTS OF INTEREST

All authors have no conflicts of interest to declare relevant to this study. Human rights statement and informed consent: This study was approved by the local ethics committee of Juntendo University, Faculty of Medicine (No. 14–103) and Sugiyama Clinic (No. 18–002). All procedures followed were in accordance with the ethical standards of the responsible committee on human experimentation and with the Helsinki Declaration of 1964 and its later amendments. All recruited women provided written informed consent. The data that support the findings of this study are available on request from the corresponding author. The data are not publicly available due to privacy or ethical restrictions. Animal studies: This article does not contain any study with animal participants that have been performed by any of the authors.

## Supporting information

Table S1
