# Supplementary material for: Novel approaches to the management of recurrent pregnancy loss: The OPTIMUM (OPtimization of Thyroid function, Thrombophilia, Immunity, and Uterine Milieu) treatment strategy
Source: Reprod Med Biol. 2021 Sep 14;20(4):524–36. doi: 10.1002/rmb2.12412 (PMC8499598; doi:10.1002/rmb2.12412)
Supplement: Supplementary file 1 — Table S1 [file RMB2-20-524-s001.docx]

**Table. S1　Clinical characteristics of women in OPTIMUM group**

|  | **< 40 years** | **≥ 40 years** | **Total** |
| --- | --- | --- | --- |
|  | **n = 67** | **n = 48** | **n = 115** |
| **Age, years, mean ± SD (range)** | 35.3 ± 3.2 (26−39) | 41.3 ± 1.2 (40−43) | 37.8 ± 3.9 (26−43) |
| **Pregnancy history, median (range)**  Gravida  Parity  No. of clinical pregnancy losses | 2 (2−7)  0 (0−1)  2 (2−7) | 3 (2−9)  0 (0−1)  2 (2−9) | 2 (2−9)  0 (0−1)  2 (2−9) |
| **AMH, ng/ml, mean ± SD** | 4.2 ± 3.3 | 2.0 ± 1.5 | 3.1 ± 2.8 |
| **Prevalence of infertility, n (%)** | 48 (71.6) | 41 (85.4) | 89 (77.4) |

SD = standard deviation; AMH = anti-Müllerian hormone
